# Restrander: rapid orientation and artefact removal for long-read cDNA data

**DOI:** 10.1093/nargab/lqad108

**Published:** 2023-12-23

**Authors:** Jakob Schuster, Matthew E Ritchie, Quentin Gouil

**Affiliations:** Epigenetics and Development Division, The Walter and Eliza Hall Institute of Medical Research, Parkville, VIC 3052, Australia; Department of Medical Biology, The University of Melbourne, Melbourne, VIC 3010, Australia; Epigenetics and Development Division, The Walter and Eliza Hall Institute of Medical Research, Parkville, VIC 3052, Australia; Department of Medical Biology, The University of Melbourne, Melbourne, VIC 3010, Australia; Epigenetics and Development Division, The Walter and Eliza Hall Institute of Medical Research, Parkville, VIC 3052, Australia; Department of Medical Biology, The University of Melbourne, Melbourne, VIC 3010, Australia

## Abstract

In transcriptomic analyses, it is helpful to keep track of the strand of the RNA molecules. However, the Oxford Nanopore long-read cDNA sequencing protocols generate reads that correspond to either the first or second-strand cDNA, therefore the strandedness of the initial transcript has to be inferred bioinformatically. Reverse transcription and PCR can also introduce artefacts which should be flagged in data pre-processing. Here we introduce *Restrander*, a lightning-fast and highly accurate tool for restranding and removing artefacts in long-read cDNA sequencing data. Thanks to its C++ implementation, *Restrander* was faster than Oxford Nanopore Technologies’ existing tool *Pychopper*, and correctly restranded more reads due to its strategy of searching for polyA/T tails in addition to primer sequences from the reverse transcription and template-switch steps. We found that restranding improved the process of visualising and exploring data, and increased the number of novel isoforms discovered by *bambu*, particularly in regions where sense and anti-sense transcripts co-occur. The artefact detection implemented in *Restrander* quantifies reads lacking the correct 5′ and 3′ ends, a useful feature in quality control for library preparation. *Restrander* is pre-configured for all major cDNA protocols, and can be customised with user-defined primers. *Restrander* is available at https://github.com/mritchielab/restrander.

## Introduction

When performing gene expression analysis via RNA-sequencing (RNA-seq), preserving information about the direction of the RNA transcript is important, especially in areas of overlapping transcription (1). With short-read sequencing, strand information is commonly preserved through selective degradation of the second strand cDNA, marked with dUTP (2). With Pacific Biosciences (PacBio) Iso-seq protocol, the polymerase read is a concatemer of alternating forward and reverse strand sequences, due to sequencing a circular template. Reads are oriented during the demultiplexing and adapter trimming step with *Lima*. With Oxford Nanopore Technologies (ONT), direct-RNA sequencing is inherently stranded since only the actual transcript is sequenced; by contrast, either the first or second-strand cDNAs may be sequenced in the direct-cDNA and PCR-cDNA protocols, resulting in both forward and reverse reads that have to be ‘restranded’ bioinformatically. Other protocols producing full-length double-stranded cDNA followed by nanopore sequencing (e.g. (3–5)) require the same restranding process.

**Figure 1.**
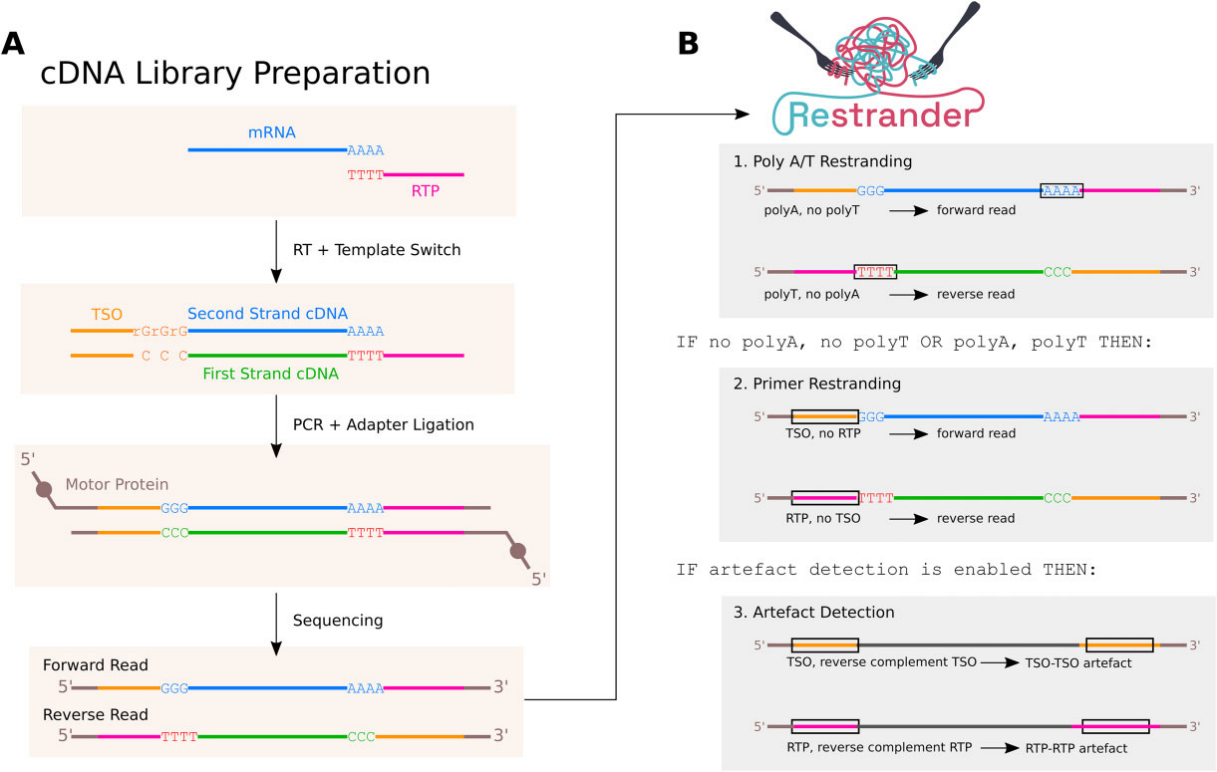
(**A**) Schematic of cDNA library preparation and sequencing. Forward reads are characterised by a lead TSO (template switch oligo) sequence, and a polyA-tail followed by the reverse complement of the reverse transcription primer (RTP). Reverse reads lead with RTP, a polyT stretch, and end with the reverse complement of the TSO. Different protocols (PCB109, PCB111, 10X Genomics, etc.) introduce variations but the principle remains the same. (**B**) The default processing pipeline used by *Restrander* to classify the direction of one read. If a read can be restranded using only the easily located polyA/T-tails, no further processing need take place. This optimisation considerably speeds up the process without significantly impacting number of incorrect classifications.

Library preparation for cDNA sequencing (Figure 1A) adds different oligonucleotide sequences on the ends of each read. The template-switching oligo (TSO, also known as Strand-Switching Primer SSP) is found on the 5’ end of forward reads, and on the 3’ end of reverse reads (as a reverse complement). The sequence of the reverse transcription primer (RTP, also known as oligo(dT) VN primer or VNP in some protocols), is conversely found at the 3’ end of forward reads (as a reverse complement) and the 5’ end of reverse reads. Thus, when reads are sequenced from the 5’ end, forward reads and reverse reads can be differentiated by the order in which the TSO and RTP appear. In addition to these primers, polyA tails are present near the end of forward reads, while complementary polyT tails are found near the start of reverse reads. Searching for these tails as well as TSO/RTP sequences provides a more confident restranding process.

A number of tools exist for read orientation, and third-party scripts have also been proposed (6). Most of these tools use either a machine-learning backend, where a pre-trained model is used to classify reads (7), or a fuzzy string search backend, where primer sequences are searched for within reads using traditional string search algorithms which allow for edit distance (8). Machine-learning backends are typically limited due to being pre-trained; if a user wants to restrand using custom primer sequences, they must retrain the model to identify these new primers. This limitation is not shared by fuzzy string search approaches, which are in principle more customisable on the user-end. *Pychopper*, developed by ONT, is the most common tool for restranding reads from fastq files from direct-cDNA or PCR-cDNA nanopore runs. It provides both a machine-learning backend based on a pre-trained hidden Markov model, and a fuzzy string search backend based on the *edlib* sequence alignment library (9). However, *Pychopper*’s Python implementation limits its computational performance, and its performance in accurately restranding reads can also be improved by parsing for polyA/T tails. Here, we introduce *Restrander*, a more powerful and flexible restranding tool based on a fuzzy string search approach. We show that *Restrander* achieves greater accuracy, correctly restranding a higher proportion of reads while being one order of magnitude faster compared to *Pychopper*. Additionally, *Restrander* classifies artefactual reads to provide useful quality control metrics and ensure only reads representing genuine RNAs are taken for downstream processing. We propose *Restrander* as an integral step of nanopore transcriptomic pipelines, between basecalling and mapping.

## Materials and methods

### Datasets

For performance testing and analysis in this paper, we used a PCR-cDNA dataset of lung adenocarcinoma cell lines (10) which included synthetic RNA spike-in (sequins (11)), deposited under accession GEO GSE172421. Reads generated with SQK-PCS109 and PBK004 kits on PromethION R9.4.1 flow cells were basecalled using *guppy* v5.1.13+b292f4d13 with parameters -c dna_r9.4.1_450bps_sup_prom.cfg--trim_strategy none--min_qscore 10--barcode_kits SQK-PCB109--do_read_splitting. For the demonstration of accuracy on ground-truth data, we focused on a subset of 30M barcode01 pass reads, corresponding to cell line NCI-H1975 and sequins *mixA*, 500K of which mapped to sequins. For speed testing, we concatenated reads from several barcodes to form fastq files of arbitrary size, producing files with 1M, 2M, 4M, 8M and 16M reads.

To test the compatibility of *Restrander* with common full-length cDNA protocols, and to evaluate its artefact detection feature, we collected datasets from: rat single B cells prepared with the NEBNext Low input/single-cell kit for NAb-seq (4) (ENA Project PRJEB51442), single-cell long-read RNA-seq from mouse muscle stem cells prepared with 10x Genomics Chromium 3′ kit (3) (GEO GSE154868) and PCR-cDNA libraries from human cell line THP-1 prepared with Oxford Nanopore Technologies SQK-PCB111 kit (ENA Project PRJEB60282).

To explore different *Restrander* parameter tunings in Supplementary Table S1, we also used a dataset of Spike-In RNA Variants (SIRV), to complement the sequin analysis. Reads generated with SQK-PCB111.24 kits on a Flongle R9.4.1 flow cells were basecalled using *guppy* v6.3.4 with parameters –trim_strategy none–barcode_kits SQK-PCB111-24–trim_adapters false–do_read_splitting and the three available models (Fast, High Accuracy, Super Accurate) to explore whether varying the basecalling accuracy would affect the optimal *Restrander* error rate. These data are deposited under ENA Project PRJEB60282.

### Alignment and transcript discovery

*minimap2* (2.17) was used to align reads to the reference genome concatenated with synthetic spike-in, with parameters -ax splice. *bambu* (1.0.3), a reference-guided transcript quantification tool for long-read RNA-seq data, was used to identify novel transcripts (12). The *bambu* parameter stranded specifies whether or not the input data is stranded. On non-stranded data, *bambu* was run with stranded=FALSE, and on restranded data *bambu* was run with stranded=TRUE. All other parameters were left at their default values. Features belonging to novel, multi-exon genes were isolated by collecting the novel genes identified and uniquely named by *bambu*, and then filtering out those with only one exon. For stranded and non-stranded data, transcripts and exons belonging to these genes were counted.

### Performance comparison

*Restrander* (1.0.0) and *Pychopper* (2.7.3) were run on Intel(R) Xeon(R) CPUs E5-2690 v4 @ 2.60GHz (Broadwell), with 12 CPUs and 32GB of memory allocated per process. The processes were timed using wall clock time as measured by the Unix time command, with default parameters. Since *Pychopper* can run on multiple threads, it was given the default 8 threads. Both of *Pychopper’s* computational backends were tested separately against *Restrander*. *Pychopper* was run with default parameters. *Restrander* was run with the default configuration for PCB109 or PCB111 data.

### Additional software

For generating the figures in this paper and performing basic analyses, R (4.2.1) (13) was used, in conjunction with the *ggplot2* (3.3.6) (14) , *tidyr* (1.2.0) (15), *patchwork* (1.1.1) (16), *cowplot* (1.1.1) (17), *Gviz* (1.40.1) (18), *ggplotify* (0.1.0) (19), *GenomicAlignments* (1.32.0) (20), *rtracklayer* (1.56.1) (21) and *RColorBrewer* (1.1-3) (22) packages.

## Results

### *Restrander* implementation

*Restrander* parses an input fastq, infers the strand of each read and prints to an output fastq. The strand of each read is recorded, and each read from the reverse strand is replaced with its reverse-complement, ensuring all reads in the output have the same orientation as the original transcripts.

In a typical cDNA-seq analysis pipeline, *Restrander* would be applied after basecalling, and before mapping. In the analysis for this paper, fastqs produced by *Guppy* were fed into *Restrander*, and then the restranded fastqs were used with *minimap2*. Only well-formed reads are included in the main output file; reads whose strand cannot be inferred are filtered out into an ‘unknown’ fastq, to be handled separately by the user. If *Restrander* is configured to detect artefacts, these artefactual reads will also be placed in the ‘unknown’ fastq.

The method used to process each read can be customised via the configuration file. Users can provide custom primer sequences and error rates, and modify parameters such as artefact detection and output verbosity to suit their needs. This enables *Restrander* to work across various protocols and contexts. Configuration files are provided for Oxford Nanopore Technologies SQK-DCS109, SQK-PCB109/PCS109, SQK-PCB111/PCS111; New England Biolabs NEBNext Single-Cell/Low Input; 10x Genomics Chromium Single-Cell 5’ and 3’ kits. *Restrander* can also be run on adapter-trimmed reads, which have polyA/T tails without primers.

./restrander input.fq.gz output.fq.gz config.json

#### Restranding process

Using the default configuration, *Restrander* first searches for polyA/T tails (Figure 1B). If the read strand cannot be conclusively inferred from this, then *Restrander* will search for TSO/RTPs. If the result is still inconclusive, the read is marked ‘unknown’. Although this initial polyA/T search speeds up restranding by about 5x overall, the user may specify to skip this step and always search for TSO/RTPs.

#### Implementation details

*Restrander* was written in C++ due to its excellent time performance over Python. The polyA/T tail classification algorithm searches for *n* consecutive A’s or T’s in the first *m* bases of the sequence. *n* = 10 and *m* = 200 by default, but both values are user-customisable. We found that if a polyA tail is not found within this hard limit *m*, it is very unlikely that one will occur at all. Thus, constraining our search to *m* does not negatively impact accuracy and ensures that extremely long sequences do not significantly slow down the restranding process. When polyA/T tail searching is unsuccessful, traditional approximate string matching will be performed to find TSOs and RTPs. By the term ‘error rate’, we refer to a Levenshtein edit distance calculated as a proportion of the length of the primer being queried over the sequence, rather than given as an absolute value. For example, searching for a 26-base pair TSO with an error rate of 0.25 would allow for an edit distance of 6, rounded down. Default error rate and search sizes were selected by parameter tuning on multiple datasets, as detailed in Supplementary Table S1.

### Demonstration on ground-truth data

To demonstrate the accuracy of *Restrander*, we analysed a PCR-cDNA dataset of human cell lines spiked-in with sequins. Sequins are synthetic transcripts with known strandedness (11). By comparing the read classifications from *Restrander* and *Pychopper* with the reads’ alignments to the sequin reference transcriptome, we determined the accuracy of both *Restrander* and *Pychopper* on real data. Each set of inputs was repeated three times.

**Figure 2.**
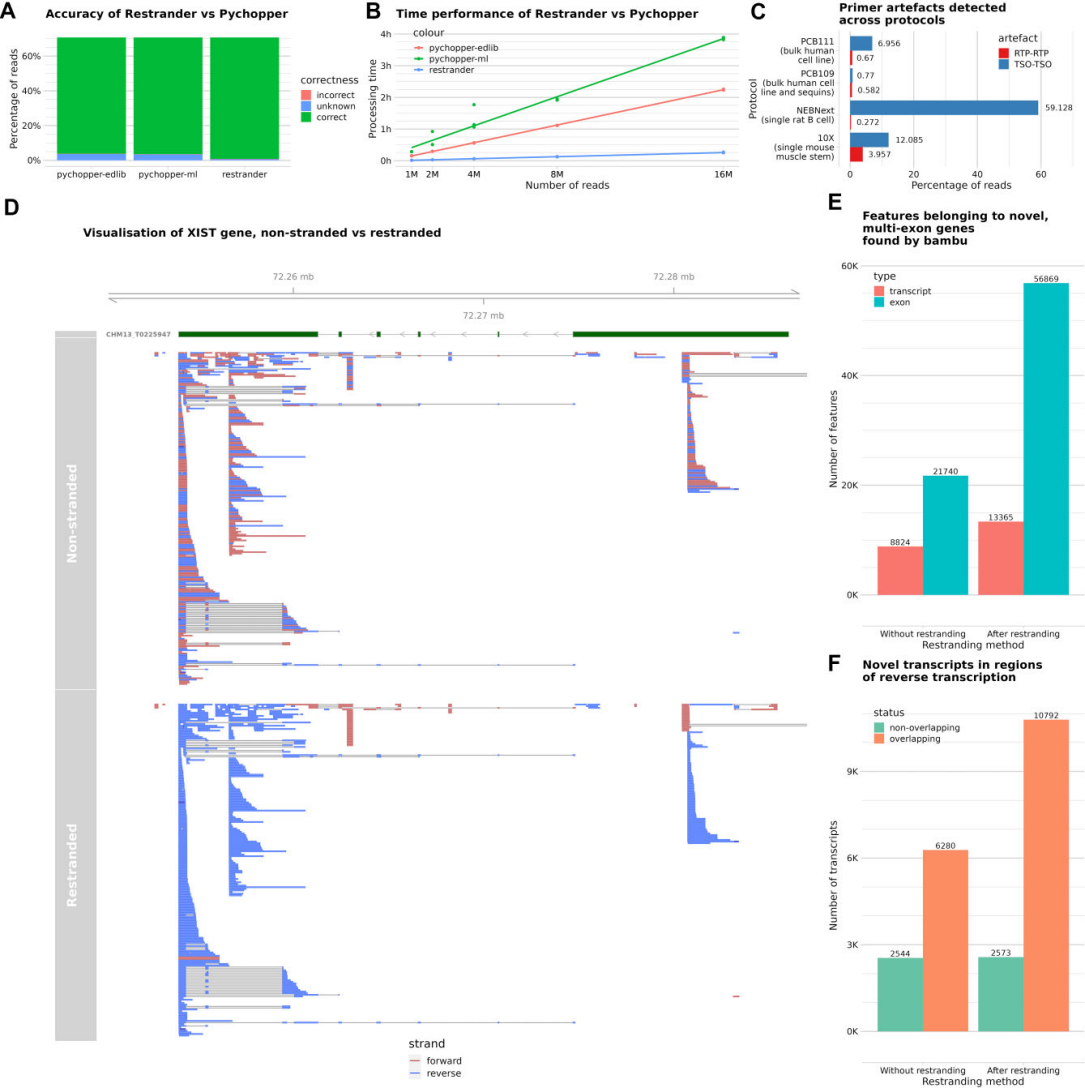
(**A**) Comparison of ground-truth accuracy of *Pychopper* and *Restrander*. (**B**) Comparison of time performance of *Pychopper* and *Restrander*. (**C**) Demonstration of artefact detection across various protocols and datasets. (**D**) Visualisation of a subsection of the *XIST* gene, with read directionality made clearer by the restranding process. (**E**) Number of features found by *bambu* on non-stranded vs restranded data, only considering features from novel, multi-exon genes. (**F**) Number of transcripts found by *bambu* originating from novel, multi-exon genes, comparing non-stranded and restranded data, and quantifying the number of transcripts which overlap with other transcripts on the opposite strand, as an indicator of sense and antisense transcript co-occurrence.

As shown in Figure 2A, *Restrander* produced the most accurate results, correctly restranding 98.84% of reads, incorrectly restranding 0.27% of reads, and leaving 0.87% of reads marked unknown. *Pychopper*’s two backends were less accurate than *Restrander*, with the machine learning and edlib backends correctly restranding 95.01% and 94.68% of reads respectively. Across a realistic dataset of 150 M reads, even a difference of 5% would result in an additional 7.5 M potentially useful reads.

*Restrander*’s superior performance was likely due to the fact that, before searching for TSO and RTPs, it searches for polyA and polyT tails, taking advantage of a useful feature overlooked by both *Pychopper* backends.

Comparing the individual reads misclassified by *Restrander* and *Pychopper*, we found that a large proportion of misclassified reads are the same across both tools (Supplementary Figure S1). Additionally, with default settings, *Restrander* categorises reads more confidently than *Pychopper*, leaving fewer unknown reads (*Restrander*’s 0.88% versus *Pychopper* ml’s 4.77% and edlib’s 5.10%) but resulting in more incorrect classifications (*Restrander*’s 0.28% versus *Pychopper* ml’s 0.22% and edlib’s 0.22%). If this is undesirable and the user would prefer to sacrifice volume of usable data for more certainty in read strandedness, they are recommended to run *Restrander* with a low error rate. This parameter and several others can be tweaked in the json configuration file.

### Speed comparison

*Restrander* runs much faster than *Pychopper*, a difference which becomes pronounced across higher volumes of data (Figure 2B). Out of the two *Pychopper* backends, the machine learning backend is the slowest, taking 3 h and 53 min to process 16 million reads. The edlib backend took only 2 h and 15 min. *Restrander* completed the same task in 15 min. On different computer architectures, performance will vary, but the relative 9× speedup of *Restrander* over *Pychopper* is expected to be consistent across different hardware contexts. In a typical PromethION flow cell sequencing run of 150M reads, it is estimated to take *Pychopper*’s edlib backend roughly 21 h to fully restrand the data, delaying downstream analysis. *Restrander* would take only 2 h and 20 min, making restranding a much less costly process. Given the volume of data involved, this step is worth optimising. This improvement is primarily due to *Restrander*’s implementation in C++, as well as some algorithmic tweaks to minimise the processing involved.

### Artefact detection

*Restrander*’s rapid identification of TSO and RTP sequences lends itself to simultaneously investigating artefacts in library preparation. These include TSO-TSO artefacts (sometimes referred to as TSO artefacts) that may constitute up to 50% of reads from single-cell cDNA libraries (5), and RTP-RTP artefacts (referred to as RTP artefacts) by second-strand cDNA priming at internal poly-T tracts in the transcript (23). These malformed reads are marked ‘unknown’ and separated from the rest of the data.

On our PCB109 data, 0.77% of reads were TSO artefacts, and 0.58% were RTP artefacts. Some datasets contained significantly more artefacts (Figure 2C). In particular, all other datasets contained more TSO artefacts than RTP artefacts; in the NEBNext single B cell data, 99.54% of the artefacts were TSO, affecting over 50% of all reads. As the datasets originate from a variety of RNA samples, we are not seeking to compare the prevalence of artefacts across different protocols, but instead demonstrate how *Restrander*’s artefact detection can be used to analyse primer-related artefacts that can be very prevalent.

### Data exploration

Restranding allows for clearer visualisation of RNA-seq data, particularly for regions where sense and antisense transcripts co-occur. To understand strange analysis results or inspect features, it is often useful to visualise data directly in IGV (24), or similar software. Restranded data can be more informative than non-stranded data for researchers to explore directly (Figure 2D).

### Impact on isoform detection

To investigate the impact of restranded vs non-stranded data on downstream processing, we analysed a lung adenocarcinoma PCR-cDNA dataset aligned to the human genome reference CHM13 using *minimap2*. Isoform detection was performed using *bambu* in either unstranded or stranded mode. Single-exon genes found by *bambu* were discarded, as they were deemed low confidence. We hypothesised that stranded data would provide *bambu* with more informative reads and allow for more reliable isoform discovery, particularly in regions of the genome with sense and anti-sense transcripts co-occurring.

Restranding allowed *bambu* to find a greater number of new features (exons and transcripts), particularly when only comparing features from novel multi-exon genes (Supplementary Figure S2); 70,234 features in stranded data versus 30,564 in non-stranded data (Figure 2E).

Of the transcripts from novel multi-exon genes, we observed that the restranded data had a greater number and higher proportion of transcripts from one strand overlapping with features found on the other (80.75% of new transcripts versus 71.12% for non-stranded data), suggesting that *bambu* found more features in areas of convergent transcription with the stranded data (Figure 2F).

## Discussion

In this paper, we present *Restrander*, a novel bioinformatic tool for quality control and transcript directionality inference of nanopore cDNA data. The software is open source and can be easily downloaded from GitHub under the MIT License. To assist users, a vignette is provided with a recommended workflow, making *Restrander* simple and easy-to-use.

*Restrander* is compatible with all common nanopore cDNA sequencing protocols, and additional configuration files can be used to accommodate new protocols and use cases. Our evaluation shows that *Restrander* outperforms *Pychopper* in both speed and accuracy in inferring transcript strand, which is critical for isoform annotation and quantification. *Restrander* offers an order of magnitude improvement in speed over *Pychopper*, and multi-threading could further enhance its performance.

Long-read sequencing technology has transformed transcriptomics, enabling the identification of full-length transcripts and alternative splicing events. However, the data generated can contain artefacts and be challenging to analyze. *Restrander* addresses this by identifying and filtering out artefacts that may affect over 50% of reads in some cases, enabling more accurate transcript quantification and splicing analysis. We also discovered that using restranded data in downstream isoform discovery via *bambu* led to the identification of more exons and transcripts, particularly in regions of convergent transcription. These novel features will require validation, which is beyond the scope of this manuscript.

We envision *Restrander* becoming a standard step in nanopore cDNA pipelines, used after basecalling and prior to mapping. By providing high-quality and stranded reads to downstream tools, *Restrander* will facilitate more accurate transcript quantification and splicing analysis.

## Supplementary Material

lqad108_Supplemental_File

## Data Availability

All supporting data is available through the following accessions: ENA Project PRJEB60282, and previously published ENA Project PRJEB51442(4), GEO GSE154868(3) and GEO GSE172421(10). Restrander is available at https://github.com/mritchielab/restrander and https://doi.org/10.5281/zenodo.10296930.
