# Supplementary material for: Restrander: rapid orientation and artefact removal for long-read cDNA data
Source: NAR Genom Bioinform. 2023 Dec 23;5(4):lqad108. doi: 10.1093/nargab/lqad108 (PMC10748469; doi:10.1093/nargab/lqad108)
Supplement: lqad108_Supplemental_File [file lqad108_supplemental_file.pdf]

Supplementary Material

A

config		sirv			sequins
		fast	hac	sup	sup
Pychopper	Edlib	69.45	71.76	72.51	93.94
	Machine Learning	69.21	70.17	70.49	94.6
Restrander, varying error rate	0.05	74.43	75.49	76.07	97.88
	0.10	75.04	75.89	76.3	98.45
	0.15	75.61	76.24	76.49	98.61
	0.20	75.83	76.38	76.56	98.71
	0.25	75.67	76.14	76.33	98.6
	0.30	72.65	72.93	73.7	97.47
	0.35	68.52	68.77	70.53	94.81
	0.40	67.91	68.33	70.22	94.75
	0.45	67.91	68.33	70.22	94.75
	0.50	67.91	68.33	70.22	94.75
	0.55	67.91	68.33	70.22	94.75
	0.60	67.91	68.33	70.22	94.75
	0.65	67.91	68.33	70.22	94.75
	0.70	67.91	68.33	70.22	94.75
	0.75	67.91	68.33	70.22	94.75
	0.80	67.91	68.33	70.22	94.75
Restrander, varying method	Skipping polyA/T	71.2	72.75	72.93	97.31
	Skipping primer	67.91	68.33	70.22	94.75
Restrander, varying search size	1000	75.33	75.8	76.5	98.53
	10000	75.21	75.72	76.46	98.5
	200	75.67	76.14	76.33	98.6
	400	75.97	76.32	76.53	98.57

B

config		sirv			sequins
		fast	hac	sup	sup
Pychopper	Edlib	100	99	98	386
	Machine Learning	411	416	414	663
Restrander, varying error rate	0.05	5	5	4	26
	0.10	5	5	5	27
	0.15	5	6	5	28
	0.20	6	6	5	30
	0.25	6	6	6	30
	0.30	7	7	6	31
	0.35	6	7	6	31
	0.40	5	6	5	29
	0.45	4	5	4	26
	0.50	4	4	4	26
	0.55	4	4	4	25
	0.60	4	4	4	24
	0.65	4	4	4	24
	0.70	4	4	4	24
	0.75	4	4	4	24
	0.80	4	4	4	24
Restrander, varying method	Skipping polyA/T	26	27	25	132
	Skipping primer	4	4	4	24
Restrander, varying search size	1000	7	7	7	38
	10000	7	8	7	39
	200	6	6	6	30
	400	7	7	6	33

Supplementary Table 1: Impact of varying *Restrander* parameters on accuracy (A) and time performance (B). The two *Pychopper* backends are also included for comparison. Where either error rate or search size have been varied, all other *Restrander* parameters have been left at their default values. Where one restranding method has been skipped, the other method has been run with default parameters. Two separate datasets were used: the PCR-cDNA dataset of lung adenocarcinoma cell lines with spike-in sequins aligned to the sequin reference transcriptome, and the pure SIRV dataset aligned to the SIRV transcripts. *Restrander* and *Pychopper* were configured for PCB111 and PCB109 primers on the SIRV and sequins data respectively.

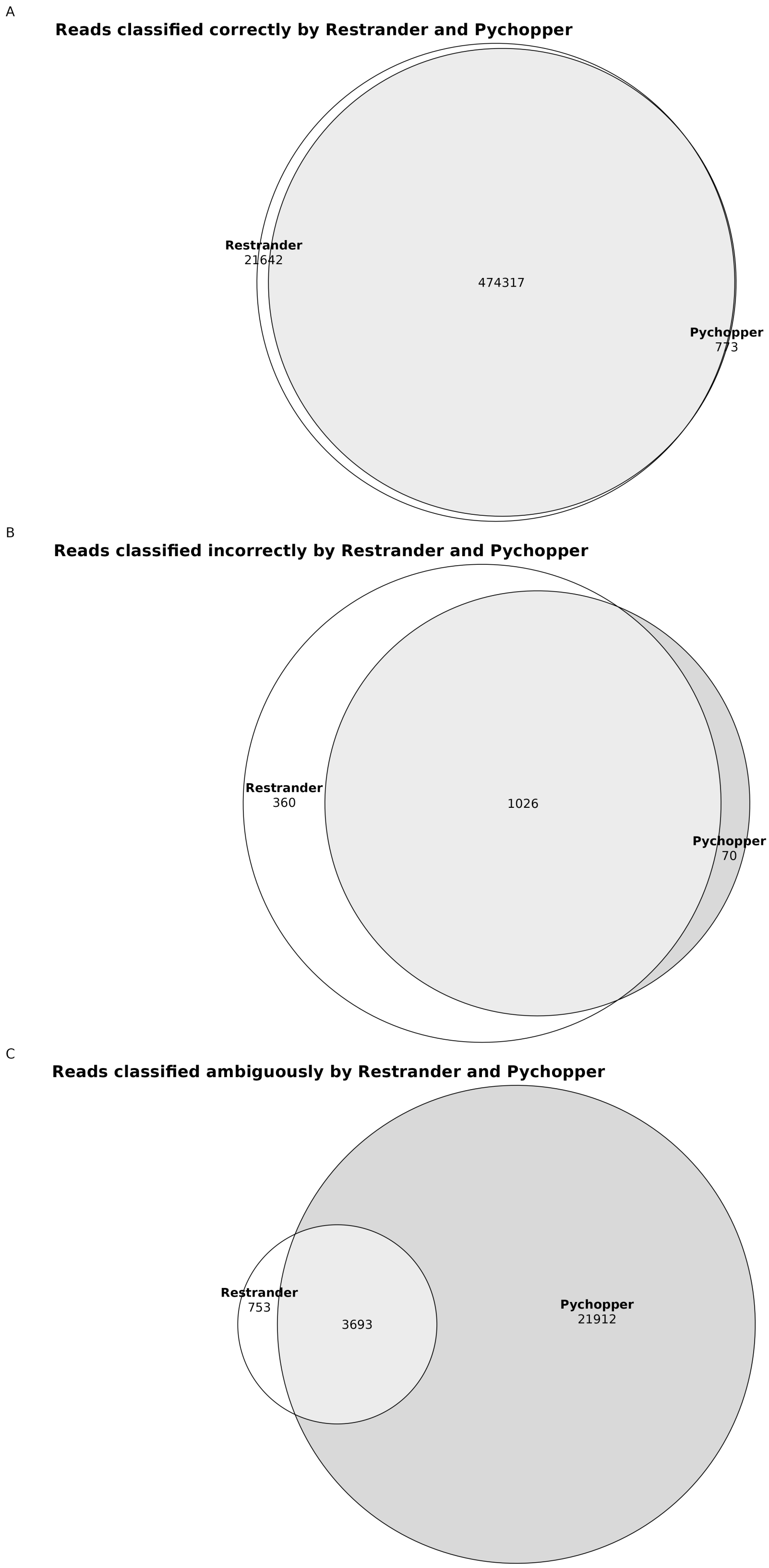

Supplementary Figure 1: Similarity between *Restrander* and *Pychopper*'s classifications of the reads aligned to the sequin reference transcriptome, using their default settings. The *edlib* backend for *Pychopper* was used, due to its slightly greater accuracy.

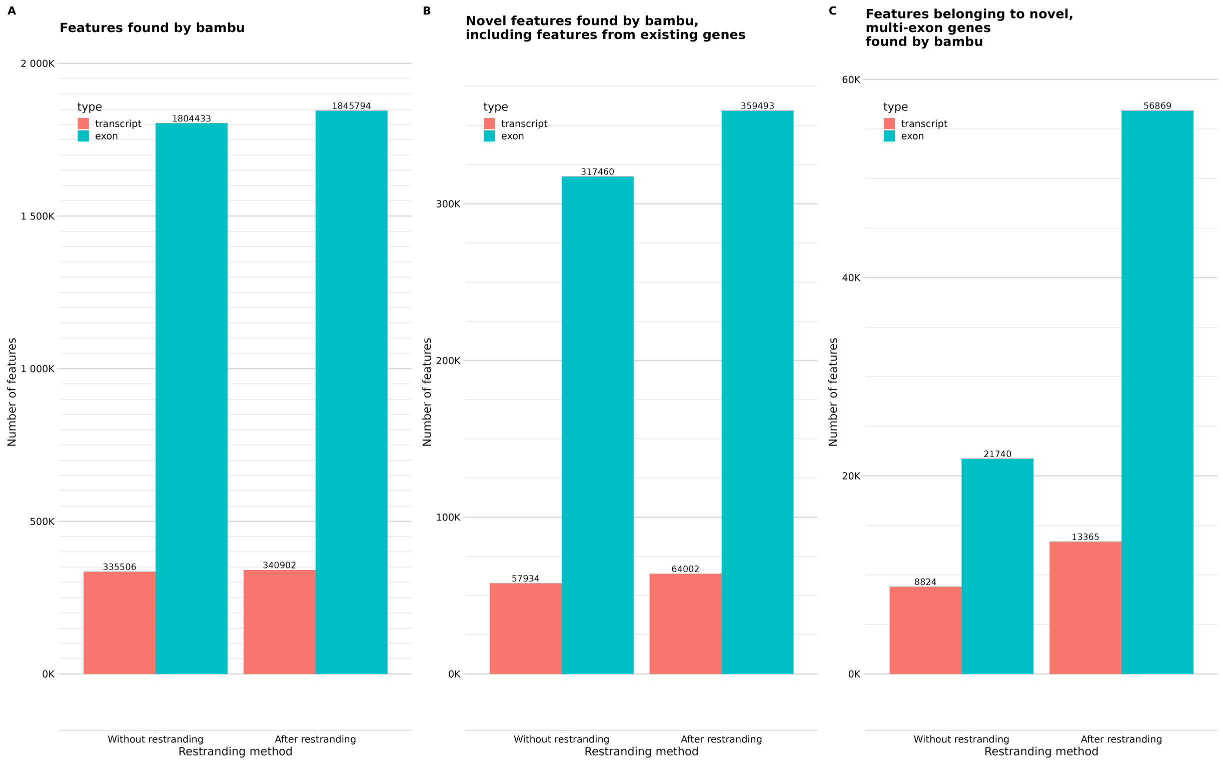

Supplementary Figure 2: Comparing the number of features found by *bambu* (A), the number of novel features found by *bambu* (B), and the number of novel features belonging to novel genes found by *bambu* (C), each with and without restranding. Each category of reads is a subset of the previous, and the difference between non-restranded and restranded data becomes increasingly pronounced across the three subsets.
